# Patient perspectives of diabetes care in primary care networks in Singapore: a mixed-methods study

**DOI:** 10.1186/s12913-023-10310-3

**Published:** 2023-12-20

**Authors:** Lay Hoon Goh, Anna Szücs, Chiew Jiat Rosalind Siah, Monica A Lazarus, E Shyong Tai, Jose M Valderas, Doris Yee Ling Young

**Affiliations:** 1 Division of Family Medicine, Yong Loo Lin School of Medicine, National University of Singapore, NUHS Tower Block Level 9, 1E Kent Ridge Road, Singapore, 119228 Singapore; 2 Alice Lee Centre for Nursing Studies, Yong Loo Lin School of Medicine, National University of Singapore, Clinical Research Centre, Block MD11, level 2, 10 Medical Drive, Singapore, 117597 Singapore; 3 Department of Medicine, Yong Loo Lin School of Medicine, National University of Singapore, NUHS Tower Block Level 10, 1E Kent Ridge Road, Singapore, 119228 Singapore

**Keywords:** Type 2 diabetes mellitus, Long-term care, Primary health care, Chronic care model, Patient assessment of chronic illness care, Integrated care

## Abstract

**Background:**

Type 2 diabetes (T2D) remains an important chronic condition worldwide requiring integrated patient-centred care as advocated by the Chronic Care Model (CCM). The Primary Care Networks (PCNs) in Singapore organise general practitioners (GPs) with nurses and care coordinators to deliver team-based care for patients with chronic conditions. This study examined the quality of care in the PCNs as defined by the CCM from the patients’ perspective.

**Methods:**

This study followed a cross-sectional convergent mixed-method design with T2D patients across three PCN types (GP-led, Group, and Cluster). The Patient Assessment of Chronic Illness Care (PACIC, range 1-5) was completed by a convenience sample of 343 patients. Multivariate linear regression was performed to estimate the associations between patient and service characteristics and PACIC summary score. Twenty-four participants were purposively recruited for interviews on the experienced care until thematic saturation was reached. Quantitative and qualitative data were collected concurrently and independently. Integration occurred during study design and data analysis using the CCM as guidance. Quantitative and qualitative results were compared side-by-side in a joint comparison table to develop key concepts supported by themes, subthemes, and patients’ quotes.

**Results:**

The PACIC mean summary score of 3.21 for 343 patients evidenced that some have received CCM consistent care in the PCNs. Being younger and spending more time with the GP were associated with higher PACIC summary scores. PACIC summary scores did not differ across PCN types. The 24 patients interviewed in the qualitative study reported receiving team-based care, nurse services, good continuity of care, as well as patient-centred care, convenient access, and affordable care. Key concepts showed that integrated care consistent with the CCM was sometimes received by patients in the PCNs. Patient activation, delivery system design/decision support, goal setting/tailoring, and problem-solving/contextual counselling were sometimes received by patients, while follow-up/coordination was generally not received.

**Conclusions:**

Patients with T2D from the Singapore Primary Care Networks received integrated care consistent with the Chronic Care Model, particularly in patient activation, delivery system design/decision support, goal setting/tailoring, and problem-solving/contextual counselling. Follow-up/coordination needed improvement to ensure higher quality of diabetes care.

**Supplementary Information:**

The online version contains supplementary material available at 10.1186/s12913-023-10310-3.

## Background

Type 2 diabetes (T2D) is a prominent chronic condition, projected to affect 783 million people worldwide by 2045 [1, 2]. Poor patient outcomes can be mitigated by providing high quality care comprising effective management and care integration [3, 4]. However, implementation of care remains a challenge for many healthcare systems poorly designed for coordinated chronic care delivery [5–11]. Primary care is able to provide integrated first-contact and accessible care for T2D patients, thanks to longitudinal and holistic interactions with patients and their families [12, 13].

Recent health policy developments in Singapore offer an excellent opportunity to examine specific primary care arrangements in the delivery of T2D care. Primary care is provided by 1,800 private general practitioner (GP) clinics and 23 public polyclinics [14]. Majority of GP clinics are single-handed practices [15], while polyclinics are large team-based practices. Patients receive government subsidies for polyclinic care, which have been extended in 2012 to certain primary care practices.

The Primary Care Networks (PCNs) formed in 2018 are networks of GPs organised into teams with nurses and care coordinators to deliver chronic disease management [16, 17] by providing ancillary services (diabetic retinal and foot screening and counselling) and care coordination. Patients with chronic conditions could use government subsidies such as Community Health Assist Scheme (CHAS) [18] and their savings (MediSave) [19], thereby reducing cash payment in the PCN clinics. There were 10 PCNs with 607 clinics in 2021, organised following three types [20]. The first type is called the GP-led PCN, formed and coordinated by partnering single-handed GPs who helm both the clinical and administrative leadership roles. There are five PCNs comprising 200 clinics under the GP-led type. The second PCN type called the Group PCN is led by two large GP corporate groups comprising 82 clinics. The third PCN type called the Cluster PCN is a partnership between single-handed GPs and the regional health clusters that included polyclinics [21]. Under the Cluster type, there are three PCNs with 325 clinics. In the Group and Cluster types, the PCN clinical leader is a GP, while the administrative leadership role is assumed by the corporate groups or cluster with whom the GPs have partnered with. The clinical leader oversees the clinical governance and development of the PCNs, while the administrative leader manages the administration in the PCNs [16]. A large majority of GP clinics in the PCNs are single-handed clinics, including those who belonged to the Group or Cluster types. After these clinics joined the PCNs, majority of them received more access to diabetes nurse services but its use could be different based on different PCN types. Only two Cluster type PCNs were organised by geographical boundaries. All other PCNs were generally located across the country. The PCNs were not organised using specific patient or clinic characteristics.

The Chronic Care Model (CCM) is a framework that supports high-quality chronic disease management that is planned, coordinated, patient-centred [22–27], and effective in improving patients’ clinical outcomes [28–31]. Over the years, the CCM has been implemented in the polyclinics for management of chronic conditions [32–34]. However, it is not known if the PCNs contain elements of CCM in providing diabetes care for their patients.

Patient engagement is an important indicator of effectiveness in chronic disease management [25]. Thus it is crucial to investigate patients’ views of care that may differ from their providers [35–37]. Yet, this is not known with respect to T2D care in the PCNs, thereby necessitating research in this area. Therefore, the aims of this study are: 1) to examine the quality of care in the PCN clinics as defined by the CCM from the patients’ perspective, and 2) to explore its determinants including the provision of care services, individual patient factors and the different PCN types.

## Methods

### Design

**Fig. 1:**
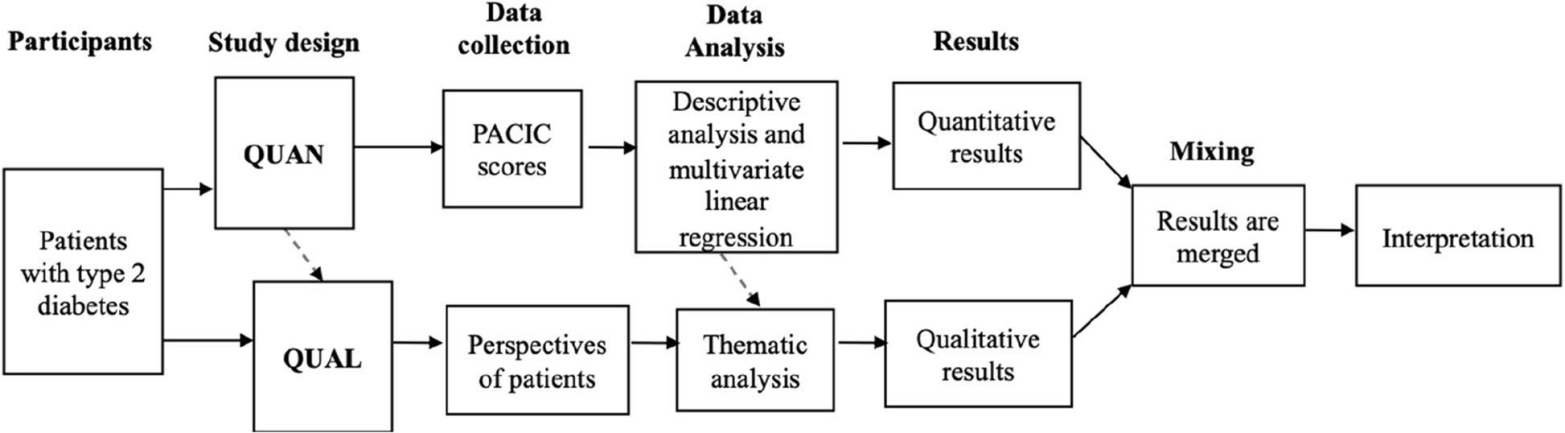
Flow diagram of studies in convergent mixed method. Footnote: QUAN (quantitative) and QUAL (qualitative) studies are equally dominant. Integration of quantitative study with qualitative study at study design and data analysis

A cross-sectional convergent mixed-method design was used for the quantitative and qualitative studies (Fig. 1). Data collection and analysis of the studies were performed concurrently and independently to support the rigorous application of the two methods [38]. Integration was performed at study design and data analysis using the CCM as guidance. The findings from the quantitative and qualitative studies were subsequently triangulated to derive integrated results and interpretations that expanded the understanding of the quantitative results [38], and provided an in-depth knowledge of the participants’ perspectives [39, 40]. This deeper understanding of the trends and patterns ensured generalizability and transferability of the results. Convergence was performed at the data summary level and not at the individual patient level.

We sent emails to the all PCN clinics explaining the study. For the PCN GPs who agreed, we recruited their patients for a survey from the clinic waiting areas or clinic lists between August 2021 and January 2022 following convenience sampling. Patients were eligible to participate in the study if they were 21 years and older, if they had a diagnosis of T2D identified by the GP, and if they had no cognitive impairment. Participants completed the survey of the quantitative arm using either paper surveys or an online link.

We purposively sampled participants from the quantitative study ensuring maximal variation by considering patients’ age, gender, ethnicity, and the PCN type of their clinic. We conducted individual interviews in English over the telephone without video function due to constraints during the COVID-19 pandemic and patients’ preference. The principal investigator LHG, a family physician with qualitative research training, conducted the interviews using a semi-structured interview guide comprising open-ended questions which were used to prompt participants to share their views on these general issues (Additional file 1). The interview questions were created in parallel with the Patient Assessment of Chronic Illness Care (PACIC) questionnaire to enable collection of contextual information related to the CCM concepts of chronic care delivery from the qualitative data. If patients did not understand any questions during the interviews, we rephrased the questions or asked follow-up questions that helped with obtaining focused answers. LHG introduced herself as a family physician who was interested to hear the participants’ views on how they received their diabetes care in the PCNs, to understand and identify any areas of care delivery that were done well and areas that needed improvement. Participants were advised that they need not answer questions that they felt uncomfortable with and that they could give their views freely without concerns that their care would be affected by what they said during the study. Interviews lasted 60 minutes on average and were audiotaped. Field notes were taken by LHG and reflexive notes written following each interview. Interviews were stopped upon reaching data saturation at the 24^th^ interview. Patients voluntarily gave their written informed consent and were reimbursed S$20 per arm for their participation. Ethics approval was obtained from the National University of Singapore Institutional Review Board (Reference Code LS-19-298).

### Measurements

A survey captured patients’ sociodemographic data and medical needs (age, gender, ethnicity, years of education, number of comorbid conditions, and use of cash payment), service-related variables (length of consultation with GP, number of nurse services received, and number of diabetes medications) and the PCN types. All information was self-reported by the patients, and not collected from clinic records.

Quality of PCN care was examined using the Patient Assessment of Chronic Illness Care (PACIC) in the English language [41]. The PACIC is a questionnaire that captures patients’ perceptions of CCM-based services that they could be expected to observe [42]. The PACIC contains 20 items reflecting the 5 subscales of patient-centred care: patient activation, delivery system design/decision support, goal setting/tailoring, problem-solving/contextual counselling, and follow-up/coordination [41] (Additional file 2). Each item is scored on a 5-point Likert scale, ranging from 1 (almost never) to 5 (almost always). Each subscale was scored by averaging responses for items within that subscale. The PACIC summary score was the average of all responses to the 20 items. Higher scores indicate the extent to which patients reported having received CCM-based services. Content validation of the PACIC was performed and resulted with minor adaptations [43, 44] (Cronbach’s alpha of 0.93). The necessary sample size for the PACIC survey was calculated to be 309 given the population of patients with T2D in the PCNs (35,667) with a margin of error of 0.1 and 95% confidence level [42, 45].

### Analysis

Bivariate analyses were performed between the PACIC summary scores and potential determinants using Pearson’s or Spearman’s correlation for continuous variables, independent T-tests for categorical binary variables, and one-way ANOVA or Kruskal-Wallis test for categorical variables with more than two categories. Variables with a *p*-value <.1 in these analyses or have clinical or conceptual relevance were entered in linear regression models in a stepwise manner, starting with the service-related variables, then including patients’ characteristics, and finally PCN types. Between each step, independent variables with *p*-value >.1 were removed from the model. Continuous variables have been standardized before entering them into the regression models. Variance inflation factors remained < 2 in all models. Statistical significance was set at *α* < 0.05. We used complete case analysis. The analyses were conducted using the Statistical Package for the Social Sciences (SPSS, Version 28, IBM Corp., Armonk, NY, USA) and R statistical software (Version 3.6.1).

Qualitative interviews were transcribed verbatim. Each transcript was independently coded by a primary coder (LHG) who identified and organised the codes into a codebook. Coding was performed using an inductive approach guided by thematic analysis [46] involving familiarisation of data, generating codes, generating themes, reviewing themes, and defining and naming themes. The transcripts were closely followed and coded multiple times to capture the original meaning of the data. Two other coders CJRS and MAL independently coded 13 and 11 transcripts respectively, to ensure concordance with the codebook. Initial codes were checked for duplicates and similarities. Similar codes were grouped under sub-themes and further aggregated into themes guided by the CCM framework. LHG, CJRS and MAL discussed the meaning of the codes, subthemes, and themes until consensus reached for the final list. Thematic saturation was achieved during analysis. All researchers reviewed and agreed on the final list of codes, subthemes and themes. The data was managed using NVivo software (1.7.1 Release), a qualitative data analysis software [47].

Using mixed methods, quantitative and qualitative results were compared side-by-side in a joint comparison table [38] using the CCM as guidance. Key concepts were developed to answer the research questions. Integration was classified as confirming or disconfirming depending on whether the quantitative or qualitative results confirmed or contradicted the key concepts, and as expanded if the results expanded the understanding of the key concepts [38].

## Results

### Characteristics of participants in quantitative study

Participants recruited were sampled from all 10 PCNs (Additional file 3). A total of 343 patients from 81 PCN GP clinics (13.3% of out of 607 clinics) participated in the quantitative study. Out of the 81 clinics, 42 (51.9%) were from the GP-led type (200 clinics in total), 15 (18.5%) from the Group type (82 clinics in total), and 24 (29.6%) from the Cluster type (325 clinics in total). Most of the participants received care at GP-led type clinics (*n*=197; 57.4%). Other participants received care from either Group type clinics (*n*=49; 14.3%), or Cluster type clinics (*n*=97; 28.3%) (Table 1). Participants in Cluster type clinics were older than in the other two types (mean 57.6 years vs 53.6 years for GP-led type and 53.5 years for Group type) (η2=0.03, 95% CI [0.003, 0.07], *p*=.006). Most participants were of Chinese ethnicity (73.2%), and the ethnicity distribution was significantly different between PCN types (Cramer’s V=0.17, *p*=.005). Participants received a median of 13 years of education (IQR 10-15, range 0-15) with no difference between PCN types. All participants had a median of one comorbid condition (IQR 1-2, range 0-4). Participants in the Cluster type had a median of two conditions as compared to the other types which had one condition. Most participants made cash payments (64.1%). More participants used cash payment in the GP-led type than in the other types (68% vs 44.9% for Group type and 66% for Cluster type) (Cramer’s V = 0.17, *p*=.009).

**Table 1:** Patients’ clinical and socio-demographic characteristics in quantitative study

	All (*N*=343)	GP-led type (*N*=197)	Group type (*N*=49)	Cluster type (*N*=97)	Effect size estimate for significant tests (95% CI)	*p-*value
Age (mean, SD)	54.7 (10.6)	53.6 (10.5)	53.5 (10.3)	57.6 (10.5)	η2 = 0.03^^^ (0.003, 0.07)	.006^**^
Gender (count/%)					NS	.052
*Male*	205 (59.8)	112 (56.9)	37 (75.5)	56 (57.7)		
*Female*	138 (40.2)	85 (43.2)	12 (24.5)	41 (42.3)		
Ethnicity (count/%)					Cramer’s V = 0.17^^^	.005^**^
*Chinese*	251 (73.2)	141 (71.6)	29 (59.2)	81 (83.5)		
*Non- Chinese*	92 (26.8)	56 (28.4)	20 (40.8)	16 (16.5)		
Years of education	13 (10-15)	13 (10-15)	13 (10-15)	13 (10-15)	NS	.680
Number of comorbid conditions	1 (1-2)	1 (1-2)	1 (1-2)	2 (1-2)	NS	.557
Cash payments, yes (count/%)	220 (64.1)	134 (68.0)	22 (44.9)	64 (66.0)	Cramer’s V = 0.17^^^	.009^**^

Bivariate analysis between PCN types and patient characteristics using one-way ANOVA or Kruskal-Wallis test for continuous variables and Chi-Square test for categorical variables

Data presented as median (IQR interquartile range) unless indicated

Legend: CI: confidence interval, NS: non-significant test, η2: Eta-squared point estimate, ^^^small effect, ^**^p<.01

Data on the length of GP consultations was missing for three participants due to participants skipping this question (Table 2). This accounted for 0.09% missing data. No other data was missing. The median length of GP consultation was 15 minutes (IQR 10-20, range 4-60). Participants used a median of two nurse services (IQR 1-4; range 0-8) across the clinics. There were significantly more nurse services used by patients in the GP-led type than in the other two types (three vs two for both Group and Cluster types) (η2=0.04, 95% CI [0.007, 0.08], *p*=.002). The median number of diabetes medications consumed by patients was one (IQR 1-2, range 0-5).

**Table 2:** Characteristics of care received by participants and PACIC scores across PCN types in quantitative study

	All (*N*=343)	GP-led type (*N*=197)	Group type (*N*=49)	Cluster type (*N*=97)	Effect size estimate for significant tests (95% CI)	*p-*value
Length of GP consultation, mins^a^	15 (10-20)	15 (10-20)	15 (10-20)	15 (10-20)	NS	.067
Nurse services, number	2 (1-4)	3 (2-4)	2 (1-3)	2 (0-3)	η2 = 0.04^^^ (0.007, 0.08)	.002^**^
Diabetes medications, number	1 (1-2)	1 (1-2)	1 (1-2)	1 (1-2)	NS	.589
PACIC scores (mean, SD)						
Summary	3.21 (0.75)	3.25 (0.78)	3.20 (0.67)	3.14 (0.71)	NS	.460
Patient Activation	3.44 (1.04)	3.49 (1.05)	3.42 (1.00)	3.35 (1.03)	NS	.524
Delivery design/decision support	3.81 (0.76)	3.81 (0.80)	3.80 (0.68)	3.82 (0.74)	NS	.986
Goal setting/tailoring	3.10 (0.83)	3.12 (0.86)	3.19 (0.76)	3.00 (0.80)	NS	.335
Problem-solving/contexual counselling	3.36 (0.93)	3.43 (0.96)	3.30 (0.83)	3.27 (0.92)	NS	.313
Follow-up/coordination	2.71 (0.90)	2.76 (0.95)	2.65 (0.81)	2.64 (0.81)	NS	.460

Bivariate analysis between PCN types and service-related variables and PACIC scores, using one-way ANOVA or Kruskal-Wallis test for continuous variables

Data presented as median (IQR interquartile range) unless indicated

^a^*n*=340

Legend: CI: confidence interval, NS: non-significant test, η2: Eta-squared point estimate, ^^^small effect, ^**^p<.01

Interpretation of PACIC scores: 1 indicates “Almost never”, 2 indicates “Generally not”, 3 indicates “Sometimes”, 4 indicates “Most of the time”, and 5 indicates “Almost always”

### Patient reported quality of care

The mean PACIC summary score was 3.21 (SD 0.75). The delivery system design/decision support subscale attained the highest mean score of 3.81 (SD 0.76), followed by patient activation subscale of 3.44 (SD 1.04), problem-solving/contextual counselling subscale of 3.36 (SD 0.93), goal setting/tailoring subscale of 3.10 (SD 0.83), and follow-up/coordination subscale of 2.71 (SD 0.90) (Table 2 and Additional file 4).

Correlation analysis between PACIC summary scores and potential determinants was performed (Additional file 5). Length of GP consultation (*r*_*s*_ = 0.19, 95% CI [0.08, 0.29], *p*<.001), number of nurse services (*r*_*s*_ = 0 .12, 95% CI [0.02, 0.23], *p*=.025), and number of diabetes medications (*r*_*s*_ = 0.15, 95% CI [0.04, 0.25], *p*=.006) were found to be positively correlated with PACIC summary scores, while age (*r* = -0.25, 95% CI [-0.35, -0.15], *p*<.001) was negatively correlated. Other variables were not significantly correlated with PACIC summary scores. Chinese ethnicity (Cohen’s d = -0.33, 95% CI [-0.57, -0.09], *p*=.009) was found to be associated with lower PACIC summary scores (Additional file 6). Other variables were not associated with PACIC summary scores.

In the multivariate analysis (Table 3), the length of GP consultations was positively associated with higher PACIC summary scores (*p*=.008) in all three models. The number of diabetes medication was positively associated with higher PACIC summary scores in the first model (*p*=.032), but not in subsequent models. Age was associated with lower PACIC scores (*p*<.001). PCN type was not associated with PACIC summary scores. All three models were significant. The service-related variables accounted for 3% of the variance in PACIC summary scores in Model 1 (adjusted R^2^=0.03). Adding patients’ characteristics in Model 2 increased the model fit (adjusted R^2^=0.09), yet adding PCN type in Model 3 did not, despite excluding non-significant co-variates (adjusted R^2^=0.08).

**Table 3:** Multivariate linear regression stepwise models testing associations with the PACIC summary scores

	Model 1		Model 2		Model 3	
	*β* (SE)	*p*-value	*β* (SE)	*p*-value	*β* (SE)	*p*-value
**Service-related**						
Length of GP consultation, minutes	0.09 (0.04)	.029*	0.09 (0.04)	.027*	0.10 (0.04)	.008**
No. of nurse services	0.07 (0.04)	.071	0.06 (0.04)	.157	Excluded	
No. of diabetes medications	0.09 (0.09)	.032*	0.07 (0.04)	.098	0.07 (0.04)	.093
**Patient characteristics**						
Age			-0.16 (0.04)	<.001***	-0.16 (0.04)	<.001***
Gender (Ref: Female)						
Male			0.04 (0.08)	.636	Excluded	
Ethnicity						
(Ref: Non-Chinese)						
Chinese			-0.15 (0.09)	.087	-0.17 (0.09)	.060
Years of education			0.01 (0.04)	.755	Excluded	
No. of comorbid conditions			0.05 (0.04)	.220	Excluded	
Cash payment (Ref: No)			-0.06 (0.08)	.490	Excluded	
**PCN types**						
PCN type (Ref: GP-led)						
Group					-0.08 (0.11)	.464
Cluster					-0.05 (0.09)	.612
	*F*(3,336)=4.93, *p*=.002, Adjusted R^2=^=0.03		*F*(9,330)=4.59, *p*<.001, Adjusted R^2^=0.09		*F*(6,333)=6.23, *p*<.001, Adjusted R^2^=0.08	

Excluded indicated independent variables that were removed with *p*>.1

*β* beta coefficient, SE Standard Error

Legends: *p<0.05, **p<0.01, ***p<0.001

### Qualitative study

The sample comprised 24 participants with fair representation from each PCN type (11 from GP-led type, six from Group type, and seven from Cluster type). The participants’ median age was 55 years (IQR 42.3-66.8, range 24-75) and an equal distribution of males (*n*=12) and females (*n*=12). Ten participants were of Chinese ethnicity. Participants had a median 13 years of education (IQR 10-15, range 10-15), and had a median of one comorbid condition (IQR 0-2, range 0-3). Eleven participants (45.8%) used cash payment. The median length of their GP consultation was 15 minutes (IQR 11.3-20, range 10-45). Participants received a median of three nurse services (IQR 2-5, range 0-7), and consumed a median of one medication (IQR 1-2, range 0-4).

### Themes and subthemes of patient experiences with diabetes care

Five themes and 18 subthemes with representative quotes covering the patients’ experiences of their diabetes care received at the PCN clinics were identified (Additional file 7). The five themes were: Theme 1 Team-based diabetes services provided by PCNs (with two subthemes), Theme 2 PCN features that were favoured by patients (with five subthemes), Theme 3 Opportunity for PCNs to collaborate with community partners (with three subthemes), Theme 4 Financial aspects of PCN care (with three subthemes), and Theme 5 Enhancement that PCNs should consider (with five subthemes).

#### Theme 1: Team-based diabetes services provided by PCNs

Patients appreciated that they received convenient nurse ancillary and care coordinator services at the clinics.

*“Dr S told me a month back, like there would be a workshop and are you interested? I said yeah, because I haven't been able to do my eye test because of this COVID measures for last year, so it’s good that I can get it done here.” (P24)*

#### Theme 2: PCN features that were favoured by patients

Patients valued that they saw the same doctor for their diabetes, highlighting how rapport brought confidence about the prescribed treatment.

*“Because I’m used to the doc, she knows my condition, what medicine to give me. Then she share so many things with me, right? How to improve my condition and advise me, check my blood test. Because she knows my condition, then I know the doctor can help me or not.” (P2)*

Patients liked that they spent sufficient time with their GPs.

*“She (the GP) doesn't talk to you in a hurry, in a hurried manner. She takes time to listen to me and yeah, just to know ... any current discomfort or anything that I need to find out from her, she's readily available for me.” (P15)*

Patients felt they were treated holistically by their GPs when discussing treatment care plans and when taught problem-solving skills.

*“He (the GP) said whether I can go for a brisk walk around the park, like running, jogging, whatever I’m at the park. If free, I can do it anytime I want, that kind of thing.” (P14)*

Patients felt actively engaged and supported by their GPs and nurses to set goals for their diabetes.

*“If the (blood glucose) is high, they (the GPs) try to check with me, what I have been doing for the past, like my diet. The way they approach the patient, something like that, which I feel comfortable.” (P6)*

Patients appreciated the convenient access to the clinics.

*“I mean it's (PCN clinic) also at the most convenient location, because ultimately, that's primary care… it's got to be easy access.” (P17)*

#### Theme 3: Opportunity for PCNs to collaborate with community partners

Patients felt that GPs and polyclinics could collaborate to deliver diabetes care by having shared medical records and polyclinic-subsidised medications.

*“Maybe medication-wise, it's possible for the GP to take the cheaper ones from the polyclinic to give [to] those who can’t really afford (the medications).” (P21)*

Most patients were not referred to community programmes such as support groups or exercise classes for their diabetes, nor were they aware of such programmes.

*“When I went to the clinic, the doctor recommended me to this diabetes association. But I live in Jurong (west of Singapore), and most of the activities, they have it at east side, like Bedok. Yeah, so it’s not convenient. After a while, I didn’t renew my membership.” (P6)*

#### Theme 4: Financial aspects of PCN care

Patients found that PCN care was affordable by using the Community Health Assist Scheme (CHAS) subsidy and their savings (MediSave).

*“I see him (the GP) every two months. So, it ranges from $60 to $130, but it is subsidised by CHAS. After my CHAS finishes, I use MediSave. So, cash payment I pay quite little, around $20 to $40, which to me is affordable.” (P23)*

However, there were worries that the medication costs would increase with time.

*“She (the GP) was doing a different brand and one box was $50. Two months of fenofibrate only added up to $11 (from the polyclinic). I can see her for monitoring for blood tests. But when it comes to taking medications every day and it’s for life. To sustain this cost, it’s just not worth it (to see the GP).” (P8)*

Patients felt that more subsidies for medications would help them stay with their GPs.

*“If they (government) reduce the price to 50% or even 25%, it actually helps a lot. Subsidise the medication, that's most expensive.” (P18)*

#### Theme 5: Enhancement that PCNs should consider

Patients identified specific areas for improvement in the PCNs.

*“But the (paper) record is very thick, like visiting this doctor for the last 10 years. Yeah, not the most efficient way to manage a patient. The cloud storage is the way towards the future. When you're traveling and something happened, at least you can send this report to the doctor there.” (P11)*

*“The nurse is always going all over (to different clinics). If you can have everything under one roof, that would be good.” (P17)*

### Integration of quantitative and qualitative results

The Patient Assessment of Chronic Illness Care (PACIC) subscales were compared with the subthemes in a joint comparison table (Table 4). There were eight subthemes that confirmed the key concepts with the quantitative results, five subthemes that disconfirmed the key concepts, three subthemes that expanded the understanding of a key concept, and five subthemes that were not covered by any PACIC scales. All subthemes were corroborated by patients’ quotes (Additional file 8).

**Table 4:** Patients’ joint comparison table showing integration analysis of quantitative and qualitative results

Integration Analysis		Quantitative results	Qualitative results
Key Concepts	Classifying the integration	PACIC subscales	Subthemes
Patient Activation was sometimes received	Confirming	Patient Activation subscale with mean score 3.44 (SD 1.04)	Subtheme 2.3 Patient-centred care received Subtheme 2.4 Engaged and supported by GPs
	Disconfirming		Subtheme 5.4 Increase self-care information in patient education
Delivery system design/decision support was sometimes received	Confirming	Delivery system design/decision support subscale with mean score 3.81 (SD 0.76)	Subtheme 1.1Nurse ancillary services provided Subtheme 2.1 Follow up by same GP Subtheme 2.2 Adequate consultation time with GPs Subtheme 2.5 Convenient access to PCN care
	Disconfirming		Subtheme 5.5 Enable more allied health services
	Expanded		Subtheme 3.1 Shared care with polyclinics Subtheme 3.2 Subsidised medications from polyclinics Subtheme 5.2 Increase access to nurse services
Goal setting/tailoring was sometimes received	Confirming	Goal setting/tailoring subscale with mean 3.10 (SD 0.83)	Subtheme 2.3 Patient-centred care received
	Disconfirming		Subtheme 3.3 Referral to community programmes
Problem-solving/contextual counselling was sometimes received	Confirming	Problem-solving/contextual counselling subscale with mean 3.36 (SD 0.93)	Subtheme 2.3 Patient-centred care received
	Disconfirming		Subtheme 2.3 Patient-centred care received
Follow-up/coordination was generally not received	Confirming	Follow-up/coordination subscale with mean 2.71 (SD 0.90)	Subtheme 1.2 Care coordination and follow-up provided Subtheme 3.3 Referral to community programmes
	Disconfirming		Subtheme 1.2 Care coordination and follow-up provided
Integration not possible	-	Not covered by any PACIC subscales	Subtheme 4.1 Affordable PCN fees Subtheme 4.2 Rising medical costs Subtheme 4.3 More government subsidises needed Subtheme 5.1Increase physical space in PCN clinics Subtheme 5.3 Increase use of electronic medical records

The eight subthemes that confirmed the key concepts were: 1.1 Nurse ancillary services provided, 1.2 Care coordination and follow-up provided, 2.1 Follow up by same GP, 2.2 Adequate consultation time with GP, 2.3 Patient-centred care received, 2.4 Engaged and supported by GPs, 2.5 Convenient access to PCN care, and 3.3 Referral to community programmes. The five disconfirming subthemes were: 1.2 Care coordination and follow-up provided, 2.3 Patient-centred care received, 3.3 Referral to community programmes, 5.4 Increase self-care information in patient education, and 5.5 Enable more allied health services. The three expanded subthemes were : 3.1 Shared care with polyclinics, 3.2 Subsidised medications from polyclinics, and 5.2 Increase access to nurse services. Lastly, the five subthemes that were not integrated were: 4.1 Affordable PCN fees, 4.2 Rising medical costs, 4.3 More government subsidises needed, 5.1 Increase physical space in PCN clinics, and 5.3 Increase use of electronic medical records.

Lastly, five key concepts were derived after integration of quantitative and qualitative results: i) Patient Activation was sometimes received, ii) Delivery system design/decision support was sometimes received, iii) Goal setting/tailoring was sometimes received, iv) Problem-solving/contextual counselling was sometimes received, and v) Follow-up/coordination was generally not received.

## Discussion

### Summary

Patients with T2D perceived that they sometimes received integrated care based on the Chronic Care Model (CCM) in the Singapore Primary Care Networks (PCNs) which varied across domains but not across PCN types. Patients were satisfied with the nurse services provided, the good continuity of care provided, having sufficient consultation time with their GPs, as well as the patient-centred approach to care involving goal setting and problem-solving, the engagement and support provided, and the convenient access to services. However, referral to community programmes and follow-up/coordination was seldom done. While patients found PCN care affordable, concerns about rising medical costs prompted suggestions to provide more medication subsidies. Specific recommendation for improvement were also made.

### Strengths and limitations

This study is the first to our knowledge to describe how patients with T2D perceive diabetes care in the Singapore PCNs. We recruited widely across the PCNs to ensure representativeness and we achieved a negligible amount of missing data. Our mixed-method design enabled us to triangulate our findings and investigate in detail the areas of care in need of improvement.

Our study has a number of limitations. The observational and cross-sectional design weakens the case for causality in the observed associations between PCN types and clinic features, and perceived quality of care in the quantitative study data. The data was obtained by patients’ self-reporting that could have recall bias [48]. Random sampling, rather than convenience sampling, would have also made the case for the generalisability of the findings more compelling. Nonetheless, our study PACIC summary scores were similar to those in studies from other countries, such as the US [41, 42, 49], Australia [50], Philippines [51], and Taiwan [52]. We were unable to obtain information about non-PCN clinics or about other non-participating patients in the PCN clinics to compare their characteristics with those of our participants. The lack of such information could have affected the generalisability of the quantitative results and the transferability of the qualitative results. Nevertheless, we were able to compare our study participants with local data. Our study had more males which concurred with the local population where more males have diabetes [53]. Additionally, the ethnicity ratio reflected the Singapore population where Chinese is the predominant ethnicity. There was a disbalance in our sampling across PCN types, which would potentially limit the generalisation of survey findings. However, adjustment for relevant variables in the stepwise regression reduced the impact of this limitation. Finally, although patients were selected based on a diagnosis of T2D, questions in the PACIC are not specific  of care for diabetes. Hence, patients' responses could also be influenced by care received for other chronic conditions. Again, triangulation of the quantitative and the qualitative results ensured a degree of specificity that might have been otherwise missing.

### Comparison with existing literature

We found that PCNs provided quality of care that varied according to the domain of interest or subscales. The subscale scores attained in our study showed similar trends with studies from Denmark [54], Philippines [55], and Switzerland [56, 57]: patient activation, delivery system design/decision support, and problem-solving/contextual counselling subscales attained higher scores, while goal setting/tailoring and follow-up/coordination subscales attained lower scores. Managing people with diabetes care involves complex delivery system designs and coordination [58, 59]. Thus, delivery system design and decision support were also the two most commonly implemented elements in clinical practice [60–62]. In contrast, goal setting/tailoring and follow-up/coordination were often under-used in chronic disease management [63].

Delivery system design/decision support attained the highest score and was confirmed by four subthemes. Firstly, patients received additional nurse ancillary services at the clinics, a feature of collaborative team-based care [20, 64–66]. Secondly, patients were satisfied that they saw the same doctor for diabetes [67–70]. Rapport built with their GPs over time could have favourably influenced their perception of care. Continuity of care with doctors was related to patient satisfaction [67, 69], lower mortality [71], greater adherence to medications [72], and reduced healthcare utilization [73]. Thirdly, patients appreciated sufficient consultation time with their doctor, a feature often lacking in primary care [74]. Our results were similar to a 2014 local study that 72% of GPs spent 15.8 minutes with their patients [15]. Sufficient consultation with physicians enabled effective communication in relation to their diabetes [75], greater patient activation [76] or involvement in their care [77–79], increased satisfaction with care [80, 81], higher levels of enablement [82], and was more patient-centred [83]. Fourthly, patients liked the convenient access to the clinics by the extended opening hours [84], the convenience of walking to the clinics from home, and the acceptable waiting time, which had comparable findings to two local studies [85, 86]. In contrast, there was a disconfirming subtheme that patients noticed the lack of allied health services in the PCNs. Although dietitians [87] and pharmacists [88] contributed to improved outcomes in the care of diabetes patients, and their roles recommended in diabetes management guidelines [89], allied health services were not well integrated in primary care [90]. Presently the nurses in the PCNs have assumed the role for nutrition advice in the absence of a dietitian.

There were three subthemes that expanded our understanding of the issues under delivery system design/decision support. The first two related to working closer to the public polyclinics. Patients suggested that polyclinics and PCN clinics have shared care or shared services since polyclinics have the ancillary nurse services under one roof on a daily basis [14], in contrast to the PCN clinics with less frequent services. Patients also proposed that subsidised medications available to polyclinic patients [34] be extended to PCN patients. Lastly, the subtheme on increasing access to the nurse services suggested that they might be presently inadequate to meet some patients’ needs [91–93].

Our patients gave high scores for the patient activation subscale which had confirming evidence from the patient-centred care demonstrated by their GPs through listening to their complaints of illness, discussing treatment plans and care goals, and problem-solving [94, 95]. Additionally, there was confirming evidence that patients felt engaged and supported by their GPs in actualising their treatment plans. However, one subtheme disconfirmed this key concept with patients requesting for more self-care information from their GPs and nurses as advised in diabetes guidelines [96]. This subtheme suggested that the present education might not be fully addressing patients’ self-management support needs.

Under problem-solving/contextual counselling, the subtheme of patient-centred care supported the key concept that patients sometimes received care in this domain in the PCNs. The confirming quote from the subtheme suggested that the GPs and nurses understood the patients’ perspectives, provided pertinent information to facilitate their autonomy in making decisions for treatment [97], and involved them in decision-making and solving problems when managing their diabetes [4, 98, 99]. In contrast, the disconfirming quote suggested that the patient sometimes disregarded their GP’s advice when the problem-solving did not consider their preferences or context.

There were care gaps identified in the goal setting/tailoring subscale. Goal setting underpins patient’s context and values, and improves their care experiences [100]. Under the confirming subtheme of patient-centred care, many patients perceived that their GPs considered their preferences in goal setting and tailored the health advice to their situations. However, no written copy of their treatment care plan was given to the patients which might have reduced the effectiveness of the goal setting. The subtheme referral to community programmes disconfirmed the key concept that advocated for tapping on community programmes to support patients in setting goals for their diabetes.

There were subthemes that confirmed that follow-up/coordination was generally not received by patients. Referrals to community programmes to support for patients after clinic visits in maintaining a healthy lifestyle might be suboptimal [101, 102] and under-utilised due to unawareness about their existence, lack of accessibility or inconvenience. However, some patients disconfirmed this key concept whereby they have received care coordination for scheduling clinic appointments from the PCN care coordinators.

Lastly, there were subthemes that were not integrated with the quantitative results but remained important to understanding patients’ perspectives about PCN care. Patients’ financial concerns about medication costs and asking for government subsidises were key factors that could influence adherence and access. A local study showed that having a new diagnosis of diabetes with other common comorbid conditions such as hypertension and hyperlipidaemia incurred the most costs for patients from new anti-diabetic medications and extra tests for monitoring and screening [103]. Medication costs for diabetes patients constituted 43% of the total direct medical burden in the US in 2017 comprising $15 billion for insulin, $15.9 billion for other antidiabetic agents, and $71.2 billion in excess use of other prescription medications [104]. Increasing patient sharing for medication costs was significantly associated with a decrease in adherence which in turn associated with poorer health outcomes [105]. In contrast, ensuring affordable medication costs could remove the barrier to access to standard diabetes care [106].

The patients’ observations that their GPs’ clinics were too small to accommodate extra nurse services have concordance from studies showing how design and layout of team spaces in primary care clinics affected how team members worked together [107, 108]. However, lack of physical space could be overcome with digital technologies and services such as telemedicine [109]. Additionally, patients’ calls that their GPs use electronic medical records instead of paper records were supported by evidence showing that chronic patients benefitted most from electronic medical records that contained decision support tools for their physicians, communication tools that informed them of their treatment, and reporting and tracking capabilities that informed them of their progress [110].

This study showed that the PCN types were not associated with the PACIC summary scores. Some aspects of quality, such as administrative and/or information technology support might not be observed by patients. Additionally, majority of the PCN clinics were single-handed practices and likely to be similar in their structure and operations despite joining different PCN types.

Lastly, we found that younger people experienced more integrated care in our study in contrast with other studies that did not find age as an association [41, 42, 50, 55, 111]. Older people with chronic conditions have increased and often unmet health and social needs [112, 113], requiring more medical care and support services [114]. Therefore, older people in this study could have perceived that more could be done for them to feel adequately supported in diabetes care. The younger adults in our study could have perceived a higher quality of care from the PCNs, based on their more positive perceptions of themselves as young people [115].

### Implications for policy, practice, and research

As patients suggested, PCN GPs might consider collaborating with polyclinics in providing shared care and subsidised medications, using more community programmes, increase clinic space for more nurse services including allied health, increase the use of electronic medical records, and having more self-care education. There was a wider concern about rising medical costs at the GP clinics and a call for more subsidises. All these issues merit consideration for improving the quality of care in PCNs in Singapore, while addressing barriers to the use of available services. Examination of the perspectives of PCN healthcare professionals is granted for corroborating and expanding the understanding of these findings in optimising healthcare delivery in Singapore for people with T2D.

## Conclusions

Patients with T2D in Singapore perceived that the PCNs provide integrated diabetes care that is consistent with the Chronic Care Model, particularly in the areas of patient activation, delivery system design/decision support, goal setting/tailoring, and problem-solving/contextual counselling. Follow-up/coordination will benefit from additional efforts.

### Supplementary Information

**Additional file 1.** Interview guide.

**Additional file 2.** Definitions of PACIC Subscale Constructs.

**Additional file 3.** Sampling strategy for patients in quantitative study.

**Additional file 4.** Observed scores for Patient Assessment of Chronic Illness Care (PACIC) subscales and items.

**Additional file 5.** Correlation analysis with PACIC summary scores.

**Additional file 6.** Analysis of associations with PACIC Summary Scores.

**Additional file 7.** Representative quotes from patients about diabetes care in PCNs, organised into themes and subthemes.

**Additional file 8.** Patient joint comparison table showing integration analysis, quantitative results, and qualitative results.

## Data Availability

All data generated or analysed in this study are included in the published article and under supplementary information.

## Abbreviations

ANOVA: Analysis of variance
*β*: Beta coefficient
CCM: Chronic Care Model
CHAS: Community Health Assist Scheme
CI: Confidence interval
η2: Eta-squared point estimate
GP: General Practitioner
IQR: Interquartile range
NS: Non-significant
PACIC: Patient Assessment of Chronic Illness Care
PCN: Primary Care Networks
*r*: Pearson’s correlation
*rs*: Spearman’s correlation
SD: Standard deviation
SE: Standard error
T2D: Type 2 diabetes
