# Supplementary material for: Patient perspectives of diabetes care in primary care networks in Singapore: a mixed-methods study
Source: BMC Health Serv Res. 2023 Dec 20;23:1445. doi: 10.1186/s12913-023-10310-3 (PMC10734143; doi:10.1186/s12913-023-10310-3)
Supplement: Supplementary file 1 — Additional file 1. Interview guide. [file 12913_2023_10310_MOESM1_ESM.docx]

**Additional file 1** Interview guide

1. What do you think of this new clinic set-up/arrangement when you have a nurse and a care coordinator (CC) in the clinic?
2. How was it for you when you met the nurse/CC at the clinic?
3. What do you think about the help/health advice you got from the nurse/CC?
4. Any issues you observed/experienced in the clinic with the nurse/CC?
5. What are some solutions you have to solve any issues you experienced?
6. What do you think can improve the way you are receiving care in this clinic?
7. Did you observe how did your GP work with the nurse/CC?
8. Did you observe how they communicate with one another?
9. Did you observe any changes in the way the clinic or your GP worked since the clinic had the nurse/CC?
10. What is your experience when receiving care at the GP clinics? Can you see the GP when you need to?
11. How does the GP involve you in your diabetes care?
12. How is the follow-up after your clinic visit?
13. How do you find the medical fees at the GP clinics?
14. Did your GP or nurse refer you to any community programmes to keep fit or manage your diabetes?
15. Are there ways that the GPs can work with the polyclinics to care for people with diabetes?

General Questions for Participants:

1. What are the reasons for your views?
2. What are some solutions you can propose to overcome the barriers for PCN?
3. What are some solutions you can propose to strengthen the facilitators for PCN?
4. How is the team working together in the clinic?
