# Supplementary material for: Patient perspectives of diabetes care in primary care networks in Singapore: a mixed-methods study
Source: BMC Health Serv Res. 2023 Dec 20;23:1445. doi: 10.1186/s12913-023-10310-3 (PMC10734143; doi:10.1186/s12913-023-10310-3)
Supplement: Supplementary file 2 — Additional file 2. Definitions of PACIC Subscale Constructs. [file 12913_2023_10310_MOESM2_ESM.docx]

Additional file 2 Definitions of PACIC Subscale Constructs

| **Subscale** | **Definition** |
| --- | --- |
| Patient Activation  (Items 1-3) | Actions that solicit patient input and involvement in decision-making |
| Delivery System Design/ Decision Support  (Items 4-6) | Actions that organize care and provide information to patients to enhance their understanding of care |
| Goal Setting/Tailoring  (Items 7-11) | Acquiring information for and setting of specific, collaborative goals |
| Problem-Solving/ Contextual Counselling  (Items 12-15) | Considering potential barriers and the patient’s social and cultural environment in making treatment plans |
| Follow-up/Coordination  (Items 16-20) | Arranging care that extends and reinforces office-based treatment, and making proactive contact with patients to assess progress and coordinate care |
