# Supplementary material for: Patient perspectives of diabetes care in primary care networks in Singapore: a mixed-methods study
Source: BMC Health Serv Res. 2023 Dec 20;23:1445. doi: 10.1186/s12913-023-10310-3 (PMC10734143; doi:10.1186/s12913-023-10310-3)
Supplement: Supplementary file 3 — Additional file 3. Sampling strategy for patients in quantitative study. [file 12913_2023_10310_MOESM3_ESM.docx]

**Additional file 3:** Sampling strategy for patients in quantitative study

| **PCN type** | **Anonymised names of PCNs** | **Number of clinics in July 2021** | **Number of clinics where recruited patients were from** | **Number of patients recruited** |
| --- | --- | --- | --- | --- |
| GP-led | A | 28 | 2 | 15 |
|  | B | 50 | 12 | 53 |
|  | C | 44 | 14 | 56 |
|  | D | 37 | 11 | 61 |
|  | E | 41 | 3 | 12 |
|  | Total | 200 | 42 | 197 |
| Group | F | 38 | 6 | 24 |
|  | G | 44 | 9 | 25 |
|  | Total | 82 | 15 | 49 |
| Cluster | H | 70 | 8 | 32 |
|  | I | 93 | 9 | 52 |
|  | J | 162 | 7 | 13 |
|  | Total | 325 | 24 | 97 |
|  | Grand total | 607 | 81 | 343 |
