# Supplementary material for: Patient perspectives of diabetes care in primary care networks in Singapore: a mixed-methods study
Source: BMC Health Serv Res. 2023 Dec 20;23:1445. doi: 10.1186/s12913-023-10310-3 (PMC10734143; doi:10.1186/s12913-023-10310-3)
Supplement: Supplementary file 4 — Additional file 4. Observed scores for Patient Assessment of Chronic Illness Care (PACIC) subscales and items. [file 12913_2023_10310_MOESM4_ESM.docx]

**Additional file 4** Observed scores for Patient Assessment of Chronic Illness Care (PACIC) subscales and items

| **Subscale** | **Mean (SD)** | **Item** | | **Mean (SD)** |
| --- | --- | --- | --- | --- |
| Patient Activation | 3.44 (1.04) | *1* | Asked for my ideas when we made a treatment plan | 3.32 (1.19) |
|  |  | *2* | Given choices about treatment to think about | 3.37 (1.20) |
|  |  | *3* | Asked to talk about any problems with medicines or their effects | 3.63 (1.15) |
| Delivery system design/  Decision support | 3.81 (0.76) | *4* | Given a list of written things I should do to improve my health | 3.14 (1.20) |
|  |  | *5* | Satisfied that my diabetes care in the clinic was well-organised | 4.37 (0.71) |
|  |  | *6* | Shown what I did to take care of my diabetes influenced my diabetes condition | 3.92 (0.95) |
| Goal setting/ Tailoring | 3.10 (0.83) | *7* | Asked to talk about my goals in caring for my diabetes | 3.53 (1.07) |
|  |  | *8* | Helped to set specific goals to improve my eating or exercise | 3.69 (1.07) |
|  |  | *9* | Given a copy of my treatment plan | 2.82 (1.27) |
|  |  | *10* | Encouraged to go to a specific group or class (e.g., support group) to help me cope with my diabetes | 2.05 (1.03) |
|  |  | *11* | Asked questions, either directly or on a survey, about my health habits (e.g., if I smoke or exercise) | 3.42 (1.16) |
| Problem-solving/ Contextual counselling | 3.36 (0.93) | *12* | Sure that my doctor or nurse thought about my values, my beliefs and my traditions when they recommended treatments to me | 3.89 (1.07) |
|  |  | *13* | Helped to make a treatment plan (medical care plan for my diabetes) that I could do in my daily life | 3.52 (1.19) |
|  |  | *14* | Helped to plan ahead so I could take care of my diabetes even in hard times (e.g., when there are family, money or work problems) | 2.95 (1.27) |
|  |  | *15* | Asked how my diabetes affects my life | 3.09 (1.17) |
| Follow-up/ Coordination | 2.71 (0.90) | *16* | Contacted after a clinic visit to see how things were going | 2.65 (1.26) |
|  |  | *17* | Encouraged to attend programs in the community (e.g., exercise program) that could help me | 2.39 (1.14) |
|  |  | *18* | Referred to a dietitian or nurse | 2.61 (1.26) |
|  |  | *19* | Told how my visits with other types of doctors, like the eye doctor, hospital specialist or surgeon, helped my diabetes treatment | 3.08 (1.21) |
|  |  | *20* | Asked how my visits with other doctors were going | 2.83 (1.24) |

**Footnotes**: Interpretation of PACIC scores: 1 indicates “Almost never”, 2 indicates “Generally not”, 3 indicates “Sometimes”, 4 indicates “Most of the time”, and 5 indicates “Almost always”
