# Supplementary material for: Patient perspectives of diabetes care in primary care networks in Singapore: a mixed-methods study
Source: BMC Health Serv Res. 2023 Dec 20;23:1445. doi: 10.1186/s12913-023-10310-3 (PMC10734143; doi:10.1186/s12913-023-10310-3)
Supplement: Supplementary file 5 — Additional file 5. Correlation analysis with PACIC summary scores. [file 12913_2023_10310_MOESM5_ESM.docx]

**Additional file 5** Correlation analysis with PACIC summary scores

| **Patient characteristics (n=343)** | **Correlation**  **coefficient** | **95% CI** | ***p-*value** |
| --- | --- | --- | --- |
| Age, years | *r* = -0.25^^^ | -0.35, -0.15 | <.001*** |
| Years of education, years | *r_s_* = 0.07^^^ | -0.04, 0.18 | .214 |
| Number of co-morbid conditions | *r_s_* = 0.02^^^ | -0.06, 0.15 | .695 |
| Length of GP consultation, minutes (n=340) | *r_s_* = 0.19^^^ | 0.08, 0.29 | <.001*** |
| Number of nurse services received | *r_s_* = 0.12^^^ | 0.02, 0.23 | .025* |
| Number of diabetes medications | *r_s_* = 0.15^^^ | 0.04, 0.25 | .006** |

Legend: CI: confidence interval, *r*: Pearson correlation, *r_s_*: Spearman’s Rank Correlation,

^^^ small effect, **p*<.05, ***p<*.01, ****p*<.001
