# Supplementary material for: Patient perspectives of diabetes care in primary care networks in Singapore: a mixed-methods study
Source: BMC Health Serv Res. 2023 Dec 20;23:1445. doi: 10.1186/s12913-023-10310-3 (PMC10734143; doi:10.1186/s12913-023-10310-3)
Supplement: Supplementary file 6 — Additional file 6. Analysis of associations with PACIC Summary Scores. [file 12913_2023_10310_MOESM6_ESM.docx]

**Additional file 6** Analysis of associations with PACIC Summary Scores

| **Patient characteristics**  **(n=343)** | **PACIC Summary Scores**  **Mean (SD)** | **Effect size estimate for significant tests (95% CI)** | ***p-*value** |
| --- | --- | --- | --- |
| Gender (Ref: Female) |  | NS | .289 |
| Female (n=138) | 3.16 (0.72) |  |  |
| Male (n=205) | 3.25 (0.76) |  |  |
| Ethnicity (Ref: Non-Chinese) |  | Cohen’s d = -0.33^^^^ (-0.57, -0.09) | .009** |
| Non-Chinese (n=92) | 3.39 (0.78) |  |  |
| Chinese (n=251) | 3.15 (0.73) |  |  |
| Cash payments (Ref: No) |  | NS | .610 |
| No (n=123) | 3.24 (0.76) |  |  |
| Yes (n=220) | 3.20 (0.74) |  |  |
| PCN type (Ref: GP-led) |  | NS | .460 |
| GP-led (n=197) | 3.25 (0.78) |  |  |
| Group (n=49) | 3.20 (0.67) |  |  |
| Cluster (n=97) | 3.14 (0.71) |  |  |

Legend: CI: confidence interval, NS: non-significant test, ^^^^moderate effect, **p<.01
