# Supplementary material for: Patient perspectives of diabetes care in primary care networks in Singapore: a mixed-methods study
Source: BMC Health Serv Res. 2023 Dec 20;23:1445. doi: 10.1186/s12913-023-10310-3 (PMC10734143; doi:10.1186/s12913-023-10310-3)
Supplement: Supplementary file 7 — Additional file 7. Representative quotes from patients about diabetes care in PCNs, organised into themes and subthemes. [file 12913_2023_10310_MOESM7_ESM.docx]

**Additional file 7** Representative quotes from patients about diabetes care in PCNs, organised into themes and subthemes

| **Theme 1 Team-based diabetes services provided by PCNs** | |
| --- | --- |
| Subtheme 1.1  Nurse ancillary services provided | Quote 1.1.1  “Okay! It was very helpful. They (the nurses) were very precise. They went through every point and everything. So, it was a good session. I could understand what they were trying to tell me.” (Patient 1)  Quote 1.1.2  “On the diet side, I met her (the nurse) once or twice … they did show me the pamphlet and then advise me on what kind of food to take, and not to take, and what kind of food that will actually trigger my sugar, you know? In fact, they gave me a booklet for me to write down my (blood glucose) readings, for me to state down, yeah. Yeah, I met them for my foot check.” (Patient 6)  Quote 1.1.3  “It was like Dr S (the GP) told me like a month back, like there would be a workshop and are you interested? I said yeah, sure, let's go ahead because I haven't been able to do my eye test because of this COVID measures for last one year, so it is good that I can get it done here. I think it was good. At least I felt comfortable that I don't have to go to the hospital to get this thing done (eye and foot screening) and that I was able to get it done at the same clinic where I visit regularly.” (Patient 24) |
| Subtheme 1.2  Care coordination and follow-up provided | Quote 1.2.1  “Okay, only when I didn’t turn up or if I’m late, they (the care coordinators) will make a call. Yeah, they will call me. But if usually I just go for my appointment, they don’t call. But I receive the SMS reminder, like a few days before the appointment date. Yeah, I know they care. But sometimes I have my own reason why I need to change appointment, that kind of thing. Yeah, I’m okay they call because I know they care for me, they want me to come according to my appointment.” (Patient 6)  Quote 1.2.2  If you missed the appointment? I think…yes…she'll call me if I didn’t go.” (Patient 11)  Quote 1.2.3  “And I think for me because my mom also had diabetes. I think for older folks, it's harder because they do not know... they need more education in terms of medication consumption. There's just too many pills for them so the, so I think perhaps what that clinic can do is to… I'm not sure whether it's practical to have a consistent follow up for elderly people who are really left on their own. Like my mom was like holding packets and packets of medication and she doesn't know what to do with it because she is illiterate, she cannot read. Yeah, so I can only think of one issue that I feel more help should be given to the people like them.” (Patient 15)  Quote 1.2.4  “Yes, and also like she make sure like we have very smooth like additional tests done even if it is not XX (name of GP clinic) which is providing this service. She ensure like you know to get the clinic and the counter person, they make the appointment on behalf of us so it is quite smooth. For example, I put my younger daughter she had a knee pain. So I took her to Dr S (the GP). She saw her and then she recommended some physiotherapy, and she got in touch with one of the physiotherapy clinic in XX (name of suburb).. And I took my daughter there and again like when she made sure like all their appointments and everything is done.” (Patient 24) |
| **Theme 2 PCN features that were favoured by patients** | |
| Subtheme 2.1  Follow up by same GP | Quote 2.1.1  “Because I’m used to the doc, she (the GP) knows my condition, she knows how the thing looks, what medicine to give me. Then she shares so many things to me, right? How to improve my condition and advise me, check my blood test. The outcome is not so good, she shares with me how to improve. She asks me, okay, try not to take this type of food, try to cut down this, cut down that. I like because she's good, very good, helpful. Because she knows my condition and then I know the doctor can help me or not, that's all.” (Patient 2)    Quote 2.1.2  “I think it is good. I think the doctor (the GP) know more about me, you see. I think mostly is because I don’t want to jump doctors … I don’t want to hop here and hop there and lose track of my profile. So I think it is okay, the family clinic is quite an integrated clinic, so I got all the care there.” (Patient 4)  Quote 2.1.3  “Yes, so he becomes my GP, I consult him for when I have flu, backache anything, without asking my orthopedic (doctor) and all that. Then he will prescribe something for me. Before the pandemic I need antibiotic bad cough, running nose. If I am driving, he will certify that my eyesight is okay.” (Patient 9)  Quote 2.1.4  “No, I don't think I'll do it because I think the doctor knows your progress, your development, so based on that, I think I'll will stick to one doctor is better because the doctor can give you further advice or maybe some action to be taken if the diabetic is getting worse and etc.” (Patient 12)  Quote 2.1.5  “No, my record, everything is with the clinic. Whatever, I will go there. Because they will check all my records. They got all my records. Yes, I will go to one… let one doctor handle me. Because they got (my) record, everything they know and they know me very well. I go there 3 years already so they know what I am taking, everything and they know my family members, my sister, my son and my daughter, they know.” (Patient 13)  Quote 2.1.6 “It depends. I feel that I don’t think it’s doctor hop, lah. I feel that the patient will feel that it is a second opinion, a third opinion. And then I also feel that there is this kind of synergy, synergy between doctors and patients. If you really don't feel good with the particular doctor, no point, no point, you know. It's kind of like chronic disease, meh? No point thinking that I always need to see that particular doctor and don't feel good about seeing the doctor. I don't think that will make your condition better. We already have a condition. I just feel that it's not good to doctor hop. Okay, for second, third opinion; but then I can understand the patients' concerns and feelings.” (Patient 16) |
| Subtheme 2.2  Adequate consultation time with GPs | Quote 2.2.1  “One advantage if I see Dr C (the GP) is that I can call one day before for the blood test and the nurse will give me an appointment, the first few patients. And Dr C will take a long time, at least 20 minutes to check on me. He will take the blood test, take my BP and my weight and see what medication I need, you see.” (Patient 9)  Quote 2.2.2  “Example, she (the GP) doesn't talk to you in a hurry, in a hurried manner. She takes time to listen to you to me and yeah, and just get to know what is my... any current discomfort or anything that I need to find out from her, she's readily available for me. (Patient 15)    Quote 2.2.3  “Definitely the GP is better. They actually spend more time with you and during consultation … you know, really having time to care for ... (the patient). Like for example, recently, I went to see Dr W (the GP). He noticed that I also just took the Sinopharm (COVID-19 vaccination); he even asked me how was my reaction, whether did I have any this ... This sort of thing is important, at least even without me asking anything, he would already start to, you know, take a look.” (Patient 17) |
| Subtheme 2.3  Patient-centred care received | Quote 2.3.1  “Oh, that one they’re very good. They say for the testing, every 3 months, before my appointment. Now, I have to come back 5 months, maybe it is quite improving. Only important is to look after your diet, don’t any how eat. Because my glucose is not so good, always 7 point something. If I can try to get lower than that, try to get lower than that.” (Patient 2)  Quote 2.3.2  “Because I have known Dr N (the GP), she’s familiar with my case, so actually she makes sure that I’m taken care of. Like the flu and pneumonia shots, it’s only at her initiative. She’s kind enough to explain to me how the CHAS card works! It was after she told me that I went to apply for it. I knew that there’s a CHAS card, but I didn't think I qualify for it.” (Patient 8)  Quote 2.3.3  “He (the GP) will ask how I have been doing, whether I got try to do anything. Then he will just keep asking then try to encourage me, although I don't really follow or I try to follow but because of my work, I'm a bit too busy to do so much exercise. He said whether I can go for a brisk walk around the park, like running, jogging, whatever I’m comfortably with at the park. He recommends at least a 30-minute brisk walk around the house area. Start maybe two or three times a week would be good. If free, I can do it anytime I want, that kind of thing.” (Patient 14)  Quote 2.3.4  “I mean, I'm quite busy most of the time, and if you ask me, I don’t like to take part in this kind of activities. You know, certain exercising so I would prefer to have this … Exercise is quite boring already, so I would prefer certain types of exercise like I like and enjoy. Some people like yoga. For me, I like dancing, so I like to choose something that I like then I go and do it, then I will not give up ma. So you ask me to join this community, unless I have all this aunty friends lah. Then that go in a group then they very free lah but I’m not very free leh, I don't have all this aunty friends leh.” (Patient 16)  Quote 2.3.5  “Basically, they (younger patients) actually look for the personalised sessions. Yeah, well, because Dr G (the GP) has been with me since my teenage years, so he knows that, Okay, without rice, it's very difficult for me. I need to have rice or a rice substitute. Then to control my sugar level, he considers, Okay, what my working style is, what work I do. I’m getting exercise in my work, so that's fine.” (Patient 18)  Quote 2.3.6  “So, he's (the GP) like ...he, besides seeing me for other illnesses, he also gets involved in my diabetes, when he sees me. Yeah, so he’s like okay, ‘How’s it going? How’s your blood levels? How’s your sugar levels? How’s your blood pressure levels.’ So involved is more like - maybe 30 percent in? Like they will advise on food, advise on exercise, advise on lifestyle. Yeah, but advice and not structure, you get what I mean? He can advise but he don't force it upon the patient. So, like 30 percent, 20 percent, doing the talks, you know, very casual.” (Patient 18)  Quote 2.3.7  “OK, yeah. Education about my food intake. Because when I see Dr Q (the GP) for my diabetes, he will check on my blood sugar, right? OK, so we have a target that we set for my blood sugar. He says he really wanted to see if I can go down to 6.5 or 6.” (Patient 23)  Quote 2.3.8  “So, I tell him the other time, there is this one of the month, last year my blood sugar shoot up to 8 suddenly. Suddenly shoot up to 8 after 2 months. So, he was surprised. So, after I was thinking and he asked me this and that. And I finally remembered that I took this supplement. So, I told him the content of the supplement got this fruit, got honey all this ah. So, then he advised me maybe this is the cause of the surge in your blood sugar, because of the content of the supplement. Ok, why don’t you stop for a while, for one month, and we will test again the next round, see if we are right. So, then I did what he advised me and true enough, it's because of the supplement lah.” (Patient 23) |
| Subtheme 2.4  Engaged and supported by GPs | Quote 2.4.1  “If let’s say it’s (blood glucose) a bit high, then they (the GPs) try to check with me: what I have been doing for the past, like my diet, that kind of thing. So usually, every after festive season, my sugar level will be up again. But then, there is no feeling like I’m scared of the doctor or what. But because, they will say, “Okay, so this time can give an excuse. So it’s just after this, continue to watch your diet, right?”. Then next time, it goes back to lower a bit, you know? Sometimes it’s up, sometimes it’s down, but then there’s always a way. The way they approach the patient, something like that, which I feel comfortable.” (Patient 6)  Quote 2.4.2  “I think the HbA1c below 6.8 or something like that. Yeah, now is like about 6.5. It was like 7 plus so he (the GP) said it was a good improvement. Just keep what I’m doing or do better, you know (laughs).” (Patient 14)  Quote 2.4.3  “Okay. I can say that that he (the GP) does his part as a doctor, you know what I mean? I mean, he cares for my family you know and also every time he would, once in a while he would call me and say, hi, how am I in a personal level. So yeah, I mean this is the first time actually I have…to get this kind of doctor, you know (laughing). Yeah, it’s about my diabetes, I mean everything about my diabetes. How, what am I doing, and how was my day and all that. So mostly it's about my condition. He'll ask about how am I feeling or am I taking my medicine and so yeah.” (Patient 19)  Quote 2.4.4  “Dr C (the GP) is really nice person. She’s telling me about everything because she takes care of patient. Yeah yeah, because the consulting everything. They talk to you nicely and they take care, after that they go check the last previous three months' report also. They want to check the blood, take the blood. After that, also tell me after two months later she asked me coming to check the conditions. She ask me what's happening, what's the problems...talk to you nicely.” (Patient 20) |
| Subtheme 2.5  Convenient access to PCN care | Quote 2.5.1  “That’s why all my friends they have a regular doctor, which is convenient, you know, for night and all that. For polyclinic only opens for 8.30 to 4.30, that’s it. But what about people work in the day, they see a doctor at night ... like me, I work most of the time in the day. So this is one of the advantage. It's the timings. Lots of the people go there after work, they go in the evening, which is convenient.” (Patient 3)  Quote 2.5.2  “Yup, and I mean it's (PCN clinic) also at the most convenient location because ultimately, that's the primary care. You want to make it primary care, then it's got to be easy access. And also at the same time, that will reduce the workload of the polyclinics, rather than everybody running there.” (Patient 17)  Quote 2.5.3  “So my work hours and all doesn't affect, it's flexible. So it's easy for me to go to the GP rather than polyclinic, yeah, because polyclinic, they close quite early. Usually in the afternoon, I think 20 plus minutes (waiting time) will be my turn already, or less than that.” (Patient 21)  Quote 2.5.4  “Waiting time is perfect for me. It's never so late, not so late, not so long waiting time. Every time, it's under control because usually when I come, my diabetes visit is usually appointment-based, right? So sometimes when I come earlier to see Dr Q (the GP) and there is no patient, he also will see me early so I don’t have to really like wait unnecessarily. It's always on time.” (Patient 23) |
| **Theme 3 Opportunity for PCNs collaborate with community partners** | |
| Subtheme 3.1  Shared care with polyclinics | Quote 3.1.1  “OK, I think if the (GP) clinic is not able to do the foot (screening), I suppose the polyclinic would be able to support it.” (Patient 15)  Quote 3.1.2  “I mean, of course, they (GP clinics and polyclinics) can definitely work together. But I think it's tough because … the patients' information may not be available to share between the GP clinic, between the different polyclinics. So how are you going to get like, all the follow up records, etc. may not be in the same system. So there might be an issue. Otherwise, if you can definitely get the GP to help, let's say do some of the screening etc. that will release some of the time for the polyclinic to check other things. Maybe that may work.” (Patient 17) |
| Subtheme 3.2  Subsidised medications from polyclinics | Quote 3.2.1  “Because most of the drugs are actually the same chemical compound. It’s just the branding, it’s just different name. So yeah, like I said previously about the government subsidy? Because of the medications that the GPs take from the branded, they're always very expensive. Yeah, so you have the generic one where like they can go and get, and which are much cheaper, that's great lah.” (Patient 18)  Quote 3.2.2  “Maybe medication wise, it's possible for the GP to take the cheaper ones from the polyclinic or something, to give those who can’t really afford (the medications).” (Patient 21) |
| Subtheme 3.3  Referral to community programmes | Quote 3.3.1  “Once the doctor (the GP) asked me to go, I said, I cannot, I got something else to do. Because I look after my mother, so I cannot attend any cooking class or what, you know. Before that the doctor asked me to go, because they say – at Lakeside, someone come and teach us what to do, what to eat. But I haven’t gone, I haven’t attend any of the class.” (Patient 5)  Quote 3.3.2  “When I went to the (GP) clinic, the lady doctor, she did recommend me this diabetes association, I can’t remember the exact name. But one thing is, it’s an annual membership, then we have to pay about $10 or something, then they will send us a pamphlet; they will send us emails when they have some activities outside. But because I live in Jurong, and most of the activities, they have it at East side, like Bedok, that kind of thing. Yeah, so it’s not so convenient. Then after a while, I didn’t renew my membership.” (Patient 6)  Quote 3.3.3  “Yes, I am actually a committee member in the CC (community centre), so I am aware of the services that is available in the CC. I am quite an active participant in the line dancing and other activities, and exercise, what was advice is to walk. Because I am a member in the CC, so I am aware what activities are good for me, not from Dr A (the GP).” (Patient 7)  Quote 3.3.4  “OK, I mean there is a Wellness Centre that's near my neighbourhood but ... no, (laughs) I don't attend the places, the community care facility for diabetic people. I don't know.” (Patient 15) |
| **Theme 4 Financial aspects of PCN care** | |
| Subtheme 4.1  Affordable PCN fees | Quote 4.1.1  “No issue, no issue. So far, the Merdeka (Community Health Assist Scheme, CHAS card) can cover my medicines still. Because I don’t have many much expensive medications.” (Patient 4)  Quote 4.1.2  “But because what I'm paying now is not that expensive, you know what I mean. So I'm thinking that for me, the brand Glucophage is not that expensive over the counter at (the PCN clinic).” (Patient 16)  Quote 4.1.3  “I usually use my CPF (Central Provident Fund). That's a good thing for Dr T (the GP). For my diabetes, my high blood pressure all that, so I ask him to take out from my CHAS card and what do you call that? Yeah, my CPF, Medisave.” (Patient 19)  Quote 4.1.4  “I see him (the GP) every two months. So, it ranges from maybe $60 to $130. But it is subsidised by CHAS (Community Health Assist Scheme). And after my CHAS finishes, I use Medisave also. So, cash payment I pay quite little. Cash payment maybe around $20 to $40, $20 to $50 like that. Every 2 months, which to me is affordable, you know?” (Patient 23) |
| Subtheme 4.2  Rising medical costs | Quote 4.2.1  “The difference, yes, if you go to polyclinic is more cheaper. You know why? The medicine, everything, I compare, currently I see it's quite expensive a bit. Here (at PCN clinic) is quite expensive, the medicine more expensive. But now, the medicine is different now, only currently taking the medicine is all unsubsidised, right? If you take other medicine, for instance, not related for your sickness, for instance for leg pain, I want some ointment, I want get some eye drop, then you pay different. It is not in the, what you call, subsidised rate, no, that’s the difference.” (Patient 2)  Quote 4.2.2  “There is a huge difference. She (the GP) was doing a different brand and a box was $50. Two months of fenofibrate only added up to $11 (from the polyclinic). The one that Dr N was carrying was one that was carried only at private clinics and costs about $120 for 2 months. It was a $100 odd difference. But for a person who is not working, I couldn’t save a lot of money. Now that I am on a lot of medicines. I can see her for monitoring for blood tests. But when it comes to taking medications every day and it’s for life; until something else comes along and the condition doesn't go away, to sustain this cost and it is just not worth it (to continue seeing the GP).” (Patient 8)  Quote 4.2.3  “To me, number one, if you talk about example, number one, everybody will talk about the price of medicine.” (Patient 23) |
| Subtheme 4.3  More government subsidises needed | Quote 4.3.1  “My wish list is most probably that they can do like, how you say, subsidies, yeah subsidies. Because we are in chronic illness, it’s going to be for life already. So maybe they want to subsidies that … So basically subsidising chronic illness will be a very good start from what I feel. Even if they reduce the price to like 50% or even 25%, it actually helps a lot. Yes. Subsidise for the medication. It's the medicines that's most expensive.” (Patient 18)  Quote 4.3.2  “I mean naturally if you get a lot of help financially on your medicine, you're happier (laughing). That you will really take care of yourself, because, you know, even I finish my medicine or what ah, I still can continue with my treatment, because I have government or people to help me with my medicine. So, when you have the feeling that you are taken care of, you are happy. When you're happier, you go on with your life better, you know. Even with sickness, you will feel positive. To me, financial is quite – is very important point in medicine ah.” (Patient 23) |
| **Theme 5 Enhancements that PCNs should consider** | |
| Subtheme 5.1  Increase physical space in PCN clinics | Quote 5.1.1  “There are quite a lot of patients. The clinic is a bit small for patients to sit especially with the COVID social distancing; we have to sit outside. The clinic is facing the morning sun, it's a bit hot. I think space, yeah, space. Space can be improved, for comfort if patients are not feeling well.” (Patient 7)  Quote 5.1.2  “I think he (the GP) needs another really better office, slightly bigger office for the diabetes care (laugh), when the patients start coming in. Because his place is a bit too small. His office is fine, but because he has the diabetes care nurse who comes in to do the foot screening and the eye screening, right? Yeah, her area is kind of small.” (Patient 18)  Quote 5.1.3  “Ah, the staff also, they are very approaching also … they nicely talk to the people. Only the place a bit small. Sometimes we need to wait outside because now the COVID situation, cannot have many people inside, so need to get outside. But they still take care of us outside there, they put some table also. They come to call us also. Our turn coming, they come to call us.” (Patient 20) |
| Subtheme 5.2  Increase access to nurse services | Quote 5.2.1  “Because right now, that nurse is always going all over (to different clinics). So in terms of the last two experience I had, I had to go to different places in order to do the thing (diabetic eye screening). So if you can have everything under one roof, that would be good but I guess maybe because of availability of the dedicated nurse to be there all the time and you may not have patient seeing every minute in time. Then I'm okay with this current arrangement (going to different venues for the screening) as well.” (Patient 17)  Quote 5.2.2  “I think that would be great … because if we can consult her (the nurse) as well, you don't have to see Dr G (the GP), take up his time if he has other patients. If she's (the nurse) permanently there, at least we have someone to help us. A diabetes care nurse will be pretty good, especially for people who have wounds that heal very slowly for diabetes and all that.” (Patient 18) |
| Subtheme 5.3  Increase use of electronic medical records | Quote 5.3.1  “That part of it I think is good if all (medical records) can be connected, so that the patients don't have to be concerned with which doctor, which polyclinic or where they are going to or if they change their (mailing) address, and they've got to change doctors or something like that. If everything is accessible but of course, there might be administrative concerns and privacy concerns. Basically, of course in the national system, their advantage of Singapore is that we are small enough to actually do it if you want to. At the moment there are areas where tests can be duplicated and that adds to the cost, if you're not monitoring carefully yourself.” (Patient 10)  Quote 5.3.2  “But the record is very thick, like visiting this doctor for the last 10 years. Then you know, your docket gets bigger in the way. There's so many medical check-ups before that go across the years, how much medication was being given. Yeah, so… not the most efficient way to manage a patient. No, no reason. The cloud storage is the way towards the future, so I don't think there's a way to avoid this. If it’s a digital record and cloud storage, I think it’s okay to have this record because I think when you're traveling and something happened, then at least you can just send this report to the doctor there.” (Patient 11)  Quote 5.3.3  “No, I think it’s paper. What’s that thing? The traditional notes, they have the big ones?” (Patient 18) |
| Subtheme 5.4  Increase self-care information in patient education | Quote 5.4.1  “From the NHS (UK National Health Service), they are very busy… you can’t see a doctor. A lot of it is about self-care… you get a lot of medicines over the counter. I took responsibility for my health. A lot of it is reading and discussing with the pre-natal and post-natal groups (referring to when she was pregnant and had diabetes). The NHS website has a lot of information- symptom checker. A lot of it is about self-education, having proper websites written by specialists, people who are experts in their fields; it’s about facts and research. This is where the habit (for self-care) is from.” (Patient 8)  Quote 5.4.2  “I wish he (the GP) will tell me more things, so I am more knowledgeable. (He) only (tells me about) the tests, check my HbA1c and give me metformin. That day, he told me if my blood test is still high next time, he have to increase the dosage (of diabetic medications), that’s what he said. Besides watching my diet, I have to exercise, good for my knees and strengthen my legs. I think he should tell me more things, but he is so busy! This Dr C, I wish he will tell patients, to inform more things, about what diet and exercise.” (Patient 9)  Quote 5.4.3  “That’s the funny part (laughing), because the first time when they (the nurses) were talking to me, they found that I actually know everything they are trying to tell me more or less. Basically, I've been reading up on my own and all that, they realised that I'm quite on the ball with my condition. In fact, I even show them the app that I use to track my sugar readings every day, what tests I do and all that. In fact, one of the nurses want to download it for her husband (laughing)! So in that sense, there was communication. They took up their book and they are running through it, everything was more or less, for me it's quite standard knowledge for diabetes control and understanding diabetes, you know.” (Patient 10) |
| Subtheme 5.5  Enable more allied health services | Quote 5.5.1  “In the polyclinic, sometimes they (the doctors) ask me to talk to dietitian, they introduce a dietitian for me to talk about my diet… diet control. I'm not too sure whether they (PCN clinic) have this service or not. Of course, it will be helpful and handy if there is one.” (Patient 4)  Quote 5.5.2  “Yeah, like the foot got problem, she (the GP) will refer me to another doctor elsewhere. This particular GP might not be able to advise you all your problem… especially when it's very complex like my case.” (Patient 11)  Quote 5.5.3  “Because my foot is under podiatry at XX (name of hospital). So I never go for foot screening that time because I just went for my foot screening at XX.” (Patient 13) |
