# Supplementary material for: Patient perspectives of diabetes care in primary care networks in Singapore: a mixed-methods study
Source: BMC Health Serv Res. 2023 Dec 20;23:1445. doi: 10.1186/s12913-023-10310-3 (PMC10734143; doi:10.1186/s12913-023-10310-3)
Supplement: Supplementary file 8 — Additional file 8. Patient joint comparison table showing integration analysis, quantitative results, and qualitative results. [file 12913_2023_10310_MOESM8_ESM.docx]

**Additional file 8** Patient joint comparison table showing integration analysis, quantitative results, and qualitative results

| **Integration Analysis** | | **Quantitative results** | **Qualitative results** | |
| --- | --- | --- | --- | --- |
| **Key concepts** | **Classifying the integration** | **PACIC subscales** | **Subthemes** | **Patients’ quotes** |
| Patient Activation was  sometimes received | Confirming | Patient Activation subscale with mean score 3.44 (SD 1.04) | Subtheme 2.3  Patient-centred care received  Subtheme 2.4  Engaged and supported by GPs | Quote 2.3.3  “He (the GP) will ask how I have been doing, whether I got try to do anything. Then he will just keep asking then try to encourage me, although I don't really follow or I try to follow but because of my work, I'm a bit too busy to do so much exercise. He said whether I can go for a brisk walk around the park, like running, jogging, whatever I’m comfortably with at the park. He recommends at least a 30-minute brisk walk around the house area. Start maybe two or three times a week would be good. If free, I can do it anytime I want, that kind of thing.” (Patient 14)  Quote 2.4.3  “Okay. I can say that that he (the GP) does his part as a doctor, you know what I mean? I mean, he cares for my family you know and also he would, once in a while he would call me and say, hi, how am I in a personal level. So yeah, I mean this is the first time actually I have…to get this kind of doctor, you know (laughing). Yeah, it’s about my diabetes, I mean everything about my diabetes. How, what am I doing, and how was my day and all that. So mostly it's about my condition. He'll [the GP] ask about how am I feeling or am I taking my medicine and so yeah.” (Patient 19) |
|  | Disconfirming |  | Subtheme 5.4  Increase self-care information in patient education | Quote 5.4.2  “I wish he (the GP) will tell me more things, so I am more knowledgeable. (He) only (tells me about) the tests, check my HbA1c and give me metformin. That day, he told me if my blood test is still high next time, he has to increase the dosage, that’s what he said. Besides watching my diet, I have to exercise, good for my knees and strengthen my legs. I think he should tell me more things, but he is so busy! This Dr C (the GP), I wish he will tell patients, to inform more things, about what diet and exercise.” (Patient 9) |
| Delivery system design/  decision support was  sometimes received | Confirming | Delivery system design/  decision support  subscale with mean score 3.81 (SD 0.76) | Subtheme 1.1  Nurse ancillary services provided  Subtheme 2.1  Follow up by same GP  Subtheme 2.2 Adequate consultation time with GPs  Subtheme 2.5 Convenient access to PCN care | Quote 1.1.3  “It was like Dr S (the GP) told me like a month back, like there would be a workshop and are you interested? I said yeah, because I haven't been able to do my eye test because of this COVID measures for last one year, so it is good that I can get it done here.” (Patient 24)  Quote 2.1.1 “Because I’m used to the doc, she (the GP) knows my condition, what medicine to give me. Then she shares so many things to me, right? How to improve my condition and advise me, check my blood test. Because she knows my condition and then I know the doctor can help me or not, that's all.” (Patient 2)  Quote 2.2.2  “She (the GP) doesn't talk to you in a hurry, in a hurried manner. She takes time to listen to you to me and yeah, and just get to know what is my... any current discomfort or anything that I need to find out from her, she's readily available for me. (Patient 15)  Quote 2.5.1  “That’s why all my friends they have a regular doctor, which is convenient, you know, for night and all that. For polyclinic only opens for 8.30 to 4.30, that’s it. But what about people work in the day, they see a doctor at night. Like me, I work most of the time in the day. So this is one of the advantage. It's the timings. Lots of the people go there after work, they go in the evening, which is convenient.” (Patient 3) |
|  | Disconfirming |  | Subtheme 5.5  Enable more allied health services | Quote 5.5.1  “In the polyclinic, sometimes they (the doctors) ask me to talk to dietitian, they introduce a dietitian for me to talk about my diet… diet control. I'm not too sure whether they (PCN clinic) have this service or not. Of course, it will be helpful and handy if there is one.” (Patient 4) |
|  | Expanded |  | Subtheme 3.1  Shared care with polyclinics  Subtheme 3.2  Subsidised medications from polyclinics  Subtheme 5.2  Increase access to nurse services | Quote 3.1.1  “OK, I think if the (GP) clinic is not able to do the foot (screening), I suppose the polyclinic would be able to support it.” (Patient 15)  Quote 3.2.2  “Maybe medication wise, it's possible for the GP to take the cheaper ones from the polyclinic or something, to give those who can’t really afford (the medications).” (Patient 21)  Quote 5.2.2  “I think that would be great … because if we can consult her (the nurse) as well, you don't have to see Dr G (the GP), take up his time if he has other patients. If she's (the nurse) permanently there, at least we have someone to help us. A diabetes care nurse will be pretty good, especially for people who have wounds that heal very slowly for diabetes and all that.” (Patient 18) |
| Goal setting/  Tailoring was  sometimes received | Confirming | Goal setting/  Tailoring subscale with mean 3.10 (SD 0.83) | Subtheme 2.3  Patient-centred care received | Quote 2.3.1  “Oh, that one they’re very good. They say for the testing, every 3 months, before my appointment. Now, I have to come back 5 months, maybe it is quite improving. Only important is to look after your diet, don’t any how eat. Because my glucose is not so good, always 7 point something. If I can try to get lower than that, try to get lower than that.” (Patient 2)  Quote 2.3.7  “OK, yeah. Education about my food intake. Because when I see Dr Q (the GP) for my diabetes, he will check on my blood sugar, right? OK, so we have a target that we set for my blood sugar. He says he really wanted to see if I can go down to 6.5 or 6.” (Patient 23) |
|  | Disconfirming |  | Subtheme 3.3  Referral to community programmes | Quote 3.3.4  “OK, I mean there is a Wellness Centre that's near my neighbourhood but no, (laughs) I don't attend the places, the community care facility for diabetic people. I don't know.” (Patient 15) |
| Problem- solving/  contextual counselling was  sometimes received | Confirming | Problem-solving/  contextual counselling subscale with mean 3.36 (SD 0.93) | Subtheme 2.3  Patient-centred care received | Quote 2.3.3  “Basically, they (younger patients) actually look for the personalised sessions. Yeah, well, because Dr G (the GP) has been with me since my teenage years, so he knows that, okay, without rice, it's very difficult for me. I need to have rice or a rice substitute. Then to control my sugar level, he considers, Okay what my working style is, what work I do. I’m getting exercise in my work, so that's fine.” (Patient 18) |
|  | Disconfirming |  | Subtheme 2.3  Patient-centred care received | Quote 2.3.4  “I mean, I'm quite busy most of the time, and if you ask me, I don’t like to take part in this kind of activities. You know, certain exercising so I would prefer to have this … Exercise is quite boring already, so I would prefer certain types of exercise like I like and enjoy. Some people like yoga. For me, I like dancing, so I like to choose something that I like then I go and do it, then I will not give up ma. So you ask me to join this community, unless I have all this aunty friends lah. Then that go in a group then they very free lah but I’m not very free leh, I don't have all this aunty friends leh.” (Patient 16) |
| Follow-up/  Coordination was generally not received | Confirming | Follow-up/  Coordination subscale of mean 2.71 (SD 0.90) | Subtheme 1.2  Care coordination and follow-up provided  Subtheme 3.3  Referral to community programmes | Quote 1.2.3  “And I think for me because my mom also had diabetes. I think for older folks, it's harder because they do not know... they need more education in terms of medication consumption. There's just too many pills for them so the, so I think perhaps what that clinic can do is to… I'm not sure whether it's practical to have a consistent follow up for elderly people who are really left on their own. Like my mom was like holding packets and packets of medication and she doesn't know what to do with it because she is illiterate, she cannot read. Yeah, so I can only think of one issue that I feel more help should be given to the people like them.” (Patient 15)  Quote 3.3.1  “Once the doctor (the GP) asked me to go, I said, I cannot, I got something else to do. Because I look after my mother, so I cannot attend any cooking class or what, you know. Before that the doctor asked me to go, because they say, someone will come and teach us what to do, what to eat. But I haven’t gone, I haven’t attended any of the class.” (Patient 5) |
|  | Disconfirming |  | Subtheme 1.2  Care coordination and follow-up provided | Quote 1.2.1 “Only when I didn’t turn up or if I’m late, they (the care coordinators) will make a call. But if I go for my appointment, they don’t call. But I receive the SMS reminder, like a few days before the appointment date.” (Patient 6)  Quote 1.2.4  “Yes, and also like she make sure like we have very smooth like additional tests done even if it is not XX (name of GP clinic) which is providing this service. She ensures like you know to get the clinic and the counter person, they make the appointment on behalf of us so it is quite smooth. For example, I put my younger daughter she had a knee pain. So I took her to Dr S (the GP). She saw her and then she recommended some physiotherapy, and she got in touch with one of the physiotherapy clinic in XX (name of suburb). And I took my daughter there and again like when she made sure like all their appointments and everything is done.” (Patient 24) |
| - | - | Not covered by any PACIC subscales | Subtheme 4.1  Affordable PCN fees  Subtheme 4.2  Rising medical costs  Subtheme 4.3  More government subsidises needed  Subtheme 5.1  Increase physical space in PCN clinics  Subtheme 5.3  Increase use of electronic medical records | Quote 4.1.4  “I see him (the GP) every two months. So, it ranges from maybe $60 to $130. But it is subsidized by CHAS (Community Health Assist Scheme). And after my CHAS finishes, I use Medisave also. So, cash payment I pay quite little. Cash payment maybe around $20 to $40, $20 to $50 like that. Every 2 months, which to me is affordable, you know?” (Patient 23)  Quote 4.2.2  “She (the GP) was doing a different brand and one box was $50. Two months of fenofibrate only added up to $11 (from the polyclinic). I can see her for monitoring for blood tests. But when it comes to taking medications every day and it’s for life. To sustain this cost and it is just not worth it (to continue seeing the GP).” (Patient 8)  Quote 4.3.2  “I mean naturally if you get a lot of help financially on your medicine, you're happier (laughing). That you will really take care of yourself, because, you know, even I finish my medicine or what ah, I still can continue with my treatment, because I have government or people to help me with my medicine.” (Patient 23)  Quote 5.1.2  “He (the GP) needs a bigger office for the diabetes care (laughs) because his place is too small. The diabetes care nurse comes in to do the foot screening and the eye screening, right? Yeah, her area is kind of small.” (Patient 18)  Quote 5.3.1  “That part I think is good if all (medical records) can be connected, so that the patients don't have to be concerned with which doctor, which polyclinic or where they are going to or if they change their address, and they've got to change doctors or something like that. At the moment, there are areas where tests can be duplicated and that adds to the cost, if you're not monitoring carefully yourself.” (Patient 10) |
